# Effects of pedigree errors on the efficiency of conservation decisions

**DOI:** 10.1186/1297-9686-41-9

**Published:** 2009-01-14

**Authors:** Pieter A Oliehoek, Piter Bijma

**Affiliations:** 1 Animal Breeding and Genomic Centre, Wageningen University, Wageningen, Gelderland, the Netherlands

## Abstract

Conservation schemes often aim at increasing genetic diversity by minimizing kinship, and the best method to achieve this goal, when pedigree data is available, is to apply optimal contributions. Optimal contributions calculate contributions per animal so that the weighted average mean kinship among candidate parents is minimized. This approach assumes that pedigree data is correct and complete. However, in practice, pedigrees often contain errors: parents are recorded incorrectly or even missing. We used simulations to investigate the effect of these two types of errors on minimizing kinship. Our findings show that a low percentage of wrong parent information reduces the effect of optimal contributions. When the percentage of wrong parent information is above 15%, the population structure and type of errors, should be taken into account before applying optimal contributions. Optimal contributions based on pedigrees with missing parent information hampers conservation of genetic diversity; however, missing parent information can be corrected. It is crucial to know which animals are founders. We strongly recommend that pedigree registration include whether missing parents are either true founders or non-founders.

## Introduction

Genetic diversity within populations is necessary for adaptive capacity and avoidance of inbreeding depression on the long term. A critical fact is that small populations are at risk of losing their adaptive capacity because genetic drift constantly lowers genetic diversity. An important strategy in conservation genetics is the preservation of genetic diversity by minimizing the average mean kinship *via *the preferential breeding of genetically important, or distantly related, animals [1,2]. In theory, the most efficient method to minimize kinship is to use optimal contribution selection (OCS) [3,4], a strategy that calculates contributions so that the weighted average mean kinship among potential parents (candidates) is minimized. This strategy associates higher contributions to genetically important animals, while animals with over-represented ancestors receive lower or zero contributions.

OCS has been implemented using either complete and correct information on pedigrees [4] or a sufficient number of molecular markers per candidate [5,6]. However, in other cases, pedigree information has been erroneous, either because of missing parent information, resulting in gaps in the pedigree, or because of wrong parent information resulting in misidentified parents. In zoo populations, missing parent information is more often the rule than the exception [7], and even for many commercial domestic populations, it is well known that the recorded pedigree does not generally fully represent the true pedigree.

Wrong parentage (misidentified parents) is often not detectable without molecular markers and can be due to (1) undetected mating (such as mating by multiple males in litters), (2) misidentification of the parent, (3) interchange of young animals, (4) data entry typos, etc. Table 1 shows an overview of the occurrence of wrong parent information in the literature as revealed by genotyping data in livestock populations [8-14]. Most authors report error rates of approximately 10%. These rates are estimates and the real percentage of undetected wrong parent information might be lower or higher. For example, Bovenhuis and van Arendonk [15] have reported an estimation of the rate of wrong parent information based on milk samples around 9 to 12%. These figures do not include only true pedigree errors, but could also result from animal sampling errors and from mixing up samples during analyses. For example, Ron *et al*. [16] and Weller *et al*. [17], in studies on the same herd found different values for wrong parent information because of differences in methodology.

**Table 1:** Overview of percentage of wrong parent information.

Population	Estimated percentage	# animals	Reference
German dairy cattle	7%	805	[8]
Israeli Holstein cows	12%	6040	[17]
Israeli Holstein cows (same pop.)	6%	249	[16]
Sheep, USA (mismothering)	10%	79	[9]
Lipizzaner Hors (mismothering)	11%	212	[10]
UK dairy cattle (misfathering)	10%	568	[11]
New Zealand dairy cattle	12–15%	several studies	[12]
Sheep, New Zealand (misfathering)	1–15%	776	[13]
Dutch dairy cows (misfathering)	9–12%	10731	[15]
Sheep, USA (misfathering)	9%	120	[14]

Literature on percentage of animals with wrong parentage; percentages represent sires, dams or both

Little is known on the effects of erroneous pedigree information on the efficiency of conservation decisions. In this article, we analyze the effect of missing parent or wrong sire information on the amount of diversity conserved when OCS is applied as a conservation strategy using a Monte Carlo simulation. We have investigated the amount of diversity saved by comparing three different situations: (1) OCS based on observed pedigree (including wrong and/or missing pedigrees), (2) OCS based on true pedigrees, and (3) breeding with equal contributions, a method that requires no (pedigree) information.

## Methods

A simulation was conducted to produce 200 replicates of diploid populations with both true and observed pedigree information. True pedigrees were converted to erroneous pedigrees using two methods: (1) changing sire records, resulting in wrong sire information (WSI) and (2) setting parent records to missing, resulting in missing parent information (MPI). To understand the impact of population parameters, a panmictic standard population and deviations were simulated. For each replicate, the true kinship based on true pedigree and the observed kinship based on observed pedigree with WSI and/or MPI were calculated in the 10^th ^generation. Subsequently, effects of pedigree errors in the 10^th ^generation were assessed using statistical criteria for true and observed kinship, and by comparing saved diversity based on true versus observed kinship. Instead of evaluating the effects for only one generation, an additional breeding scheme evaluated effects over multiple generations. In all schemes, the population sizes and sex ratios varied.

### Standard population

A panmictic (random mating) population was used as the basic model. Populations were bred for 10 discrete generations from a base generation of (unrelated) founders. For each generation, 10 males and 50 females were randomly selected as parents of the next generation. Females produced an average litter of 2.5, which was a Poisson-distributed litter size. Males had a Poisson-distributed number of mates (on average 5) and the average number of progeny was 12.5. For each generation, offspring were produced using random mating and both the true and observed pedigrees were recorded. Parameters derived from observed pedigree information are indicated with '**~**' in this paper. True kinship (*f*) between individuals was calculated from the true pedigree, and observed kinship ($\tilde{f}$) was calculated from the observed pedigree using the tabular method [18]. The 10^th ^generation had a fixed number of 100 individuals (candidate parents).

### Erroneous pedigrees

#### Wrong sire information (WSI)

For each generation, observed pedigrees were created from true pedigrees by substituting 0% to 25% of the true fathers by another father taken at random from the same generation as the true father.

#### Missing parent information (MPI)

For each generation, observed pedigrees were created from true pedigrees, by setting, sires, or both parents to missing for 0% to 100% random individuals.

#### WSI and MPI combined

The combined effect of WSI and MPI was investigated by applying 0% to 100% MPI on the standard population with 10% WSI.

### Correction for missing pedigree information

Kinship can be corrected for MPI. VanRaden [19] have stated that unknown parents should be related to all other parents by twice the mean inbreeding level of the period. Instead of mean inbreeding level, the average mean kinship among parents was used.

### Analysis

For each replicate, both true and observed kinships were calculated between all pairs of individuals from the 10^th ^generation using the tabular method [18]. The effect of WSI and/or MPI was investigated by comparing true and observed kinships using two types of criteria: (1) statistical criteria and (2) a diversity criterion.

#### Statistical criteria

Three statistical criteria were used for the analysis: (1) the correlation between true and observed kinships (*ρ*), which measures the proportion of the variance in true kinship explained by observed kinship; (2) the regression coefficient of observed kinship on true kinship (*β1*), which is a measure for bias in the observed differences in kinship among pairs of individuals; and (3) the regression coefficient of true kinship on observed kinship (*β2*), which indicates whether observed kinship is an "unbiased" prediction of true kinship. In practice, the latter is important since conservation decisions are based on observed kinship and not on true values [6]. Kinship of individuals with themselves was excluded from all three statistical criteria.

#### Diversity measures

Though statistical criteria are informative, they do not directly reveal the amount of conserved genetic diversity when using observed pedigrees in practice. In addition, we applied a diversity criterion, *DS*, which evaluates the Diversity Saved when optimal contributions are based on observed pedigrees. *DS *was calculated from three underlying diversity measures, which are expressed on the scale of founder genome equivalents (*FGE*) [20]. *FGE*s are the number of equally contributing founders with no random loss of founder alleles in descendants that would be expected to produce the same genetic diversity (or kinship) as the population under study [20,21]. This scale is a natural number and easier to interpret than probabilities or percentages [22]. The three underlying diversity measures were (1) *N*_*EC*_, genetic diversity conserved when equal contributions were applied; (2) *N*_*OC*_, genetic diversity conserved when OCS were applied based on true kinship; and (3) ${\tilde{N}}_{OC}$, the genetic diversity conserved when OCS were applied based on observed kinship (hence the '**~**').

The three diversity measures *N*_*EC*_, *N*_*OC*_, and ${\tilde{N}}_{OC}$ were based on a weighted average mean kinship among candidate parents [23]. The diversity measures (*dm*) were calculated using the following Equation:

(1) $$N_{dm}=\frac{1}{2*c'Fc},$$

where **F **is a matrix of true kinships among all individuals, including kinship of individuals with themselves, and **c **is a column vector of proportional contributions of candidate parents to future generation (which were always 100 animals in the 10^th ^generation), so that sum of elements of **c **equals one [18]. By varying the contributions of individuals (**c**), average mean kinship among candidates, and thus the average mean kinship in the future generations, can be increased or decreased.

*N*_*EC *_was calculated by substituting **c **in Equation 1 with **c**_*EC*_, which is a vector of equal contributions per candidate parent, so that the sum of elements of **c**_*EC *_equals one. *N*_*EC *_is simply the average mean kinship of the current population, expressed on the scale of FGE.

*N*_*OC *_was calculated by substituting **c **in Equation 1 with **c**_*OC*_, which is an optimum contribution vector that minimizes **c'Fc**, and therefore maximizes diversity. **c**_*OC *_is given by:

(2) $$c_{OC}=\frac{F^{-1}1}{1'F^{-1}1},$$

where **1 **is a column vector of ones. When negative contributions were obtained, the most negative contribution was set to zero and vector **c**_*OC *_was recalculated until all contributions were non-negative. This method does not necessarily find the true optimal solution. True optimum was always found, however, when contributions were not fixed a [3]. *N*_*OC *_measures the diversity that could be obtained in future generations (assuming overlap) and a practical example is the selection of animals for a gene bank to reconstruct a future population.

${\tilde{N}}_{OC}$ was calculated by substituting **c **in Equation 1 with the *observed *optimum contribution vector (${\tilde{c}}_{OC}$).${\tilde{c}}_{OC}$ was calculated by substituting **F **in Equation 2 by the matrix of observed kinship ($\tilde{F}$). ${\tilde{N}}_{OC}$ measures the obtained diversity when OCS is applied on observed pedigrees.

The diversity criterion represents the fraction Diversity Saved (*DS*) by applying optimal contributions based on observed pedigree; this was calculated as follows:

(3) $$DS=\frac{{\tilde{N}}_{OC}-N_{EC}}{N_{OC}-N_{EC}}.$$

*DS *evaluates the Diversity Saved when optimal contributions were based on observed pedigrees; ${\tilde{N}}_{OC}-N_{EC}$, as a fraction of the full amount of diversity that could have been saved with optimal contributions based on true pedigree data; *N*_*OC *_– *N*_*EC*_. Equal contributions were used as a base of comparison, as this would be the logical selection method if no information on kinship is available.

Note that in practice not all the individuals can be parent, even when desired, which causes genetic drift. This could cause a setback in the genetic diversity gained for both equal contribution- as well as optimal contribution-schemes.

The 'observed *N*_*OC*_' (${\tilde{\tilde{N}}}_{OC}$) was calculated by substituting **c **and **F **in Equation 1 with ${\tilde{c}}_{OC}$ and $\tilde{F}$. Breeders only have observed pedigrees. Therefore, the true genetic diversity obtained due to optimal contributions (${\tilde{N}}_{OC}$) is not known to breeders. Hence, ${\tilde{\tilde{N}}}_{OC}$ is the genetic diversity that breeders predict to obtain, based on the observed pedigrees.

### Optimal contribution selection scheme for multiple generations

To analyze the effect of WSI and MPI on genetic diversity over multiple generations, OCS was applied as a breeding scheme. The first five generations were randomly bred like the standard population. The following five generations were bred using OCS based on observed pedigrees. Each sex contributed half the genes to the next generation. OCS were calculated including this constraint using Sonesson and Meuwissen [4]:

(4) $${\tilde{c}}_{OC}=\{(Q{\tilde{F}}^{-1}){\left[(Q{\tilde{F}}^{-1})Q'\right]}^{-1}\}1,$$

where ${\tilde{c}}_{OC}$ is a vector of proportional contributions of (*n*) selection candidates to the next generation, so that contributions of males within ${\tilde{c}}_{OC}$ equals 1/2 and contributions of females within ${\tilde{c}}_{OC}$ equals 1/2, $\tilde{F}$ is a matrix of kinship based on observed pedigrees, **1 **is a column vector of ones, and **Q **is a (2 × n) design matrix indicating sex of the selection candidates. When negative contributions were obtained, the most negative contribution was set to zero and ${\tilde{c}}_{OC}$ was recalculated until all contributions were non-negative. Next, these continuous contributions per candidate were converted into a desired number of offspring per candidate. Each generation, mating began with a randomly assigned male and female that produced progeny, until one reached its desired number of offspring. Then, another random male or female candidate was assigned to the remaining male or female in order to produce progeny until one reached its desired number of offspring. This was repeated until all selected candidates reached their desired number of offspring, and the last generation resulted in 100 individuals. ${\tilde{N}}_{OC}$, *N*_*OC *_and *N*_*EC *_were obtained by five generations of selection using Equation 4: with ${\tilde{N}}_{OC}$ selection was based on pedigrees containing errors; with *N*_*OC *_selection was based on true pedigrees; and with *N*_*EC *_selection was based on MPI of 100% (a scenario that comes close to equal contributions). Hence, *DS *was calculated by equation 3.

## Results and discussion

### Wrong sire information (WSI)

**Figure 1:**
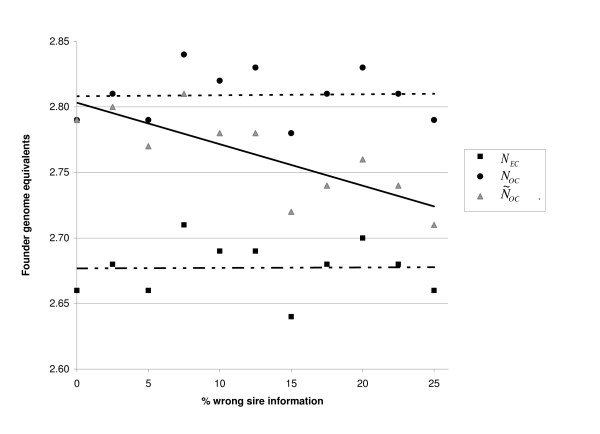
**Diversity in a panmictic population with wrong sire information**. Results are averages of 200 replicates of the standard population. Standard errors of results were 0.02. Trend lines are added for each legend entry. *N*_*EC *_is Founder genome equivalent of the average kinship (achieved by applying equal contributions). *N*_*OC *_is Founder genome equivalent of the average kinship achieved by applying optimal contributions based on true pedigrees. ${\tilde{N}}_{OC}$ is Diversity Criterion, the founder genome equivalent of the average kinship achieved by applying optimal contributions based on observed pedigrees.

Figure 1 shows diversity expressed in founder genome equivalents (FGE) of the standard population with increasing percentages of WSI in three ways: average kinship (*N*_*EC*_), optimal kinship (*N*_*OC*_) and ${\tilde{N}}_{OC}$, which is the true kinship from applying OCS on observed (possible erroneous) pedigrees. In the standard population, the average *N*_*EC *_was 2.68 and average *N*_*OC *_was 2.81, which shows that genetic diversity can be increased by applying OCS. The fluctuation of *N*_*EC*_, *N*_*OC *_and ${\tilde{N}}_{OC}$ among scenarios was due to random variation among replicates, and was equal for all three measures. As expected, ${\tilde{N}}_{OC}$ equalled *N*_*OC *_when the percentage of WSI was zero. With increasing percentage of WSI from 0% to 25%, ${\tilde{N}}_{OC}$ decreased approximately linearly.

**Figure 2:**
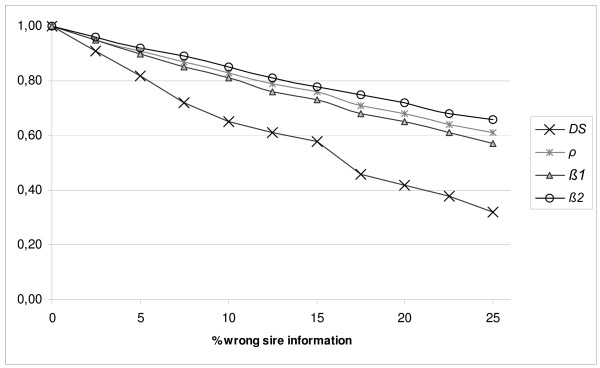
**Criteria in a panmictic population with wrong sire information**. Results are averages of 200 replicates of the standard population. Standard errors of results were 0.01 or less, except for *DS *with % wrong sire information that were higher than 15%; standard errors were 0.02.*DS *is the proportion of kinship saved by applying optimal contributions based on observed pedigrees instead of true pedigrees.*ρ *is correlation between observed kinship and true kinship.*β1 *is regression coefficient of observed kinship on true kinship.*β2 *is regression coefficient of true kinship on observed kinship.

Figure 2 shows the statistical criteria and *DS *for the same schemes as in Figure 1. Figure 2 shows that when the percentage of WSI increase, *DS*, correlation and regression (*β1 *and *β2*) decrease approximately linearly. However, *DS *decreases faster than correlation. As shown in Figure 1, *DS *follows the trend line of ${\tilde{N}}_{OC}$ and decreases approximately by 0.029 with each 1% increase of WSI. Extrapolation of results for *DS *in the standard population indicates that, on average, *DS *would be zero at a WSI of approximately 35%. In other words, from 0 to 35% WSI, when OCS is applied, diversity is on average still higher than would be the case if equal contributions were applied (*N*_*EC*_).

Simulations with larger population sizes or differences in sex ratio showed the same trend for *β1*, *β2*, *ρ *and *DS *as the standard population (results not shown). The slope of *DS *was less than when sex ratio was higher. For example, with a 1:1 sex ratio, *DS *decreases by about 0.022 with each 1% increase of WSI, and *DS *would be zero at approximately 45% WSI.

**Figure 3:**
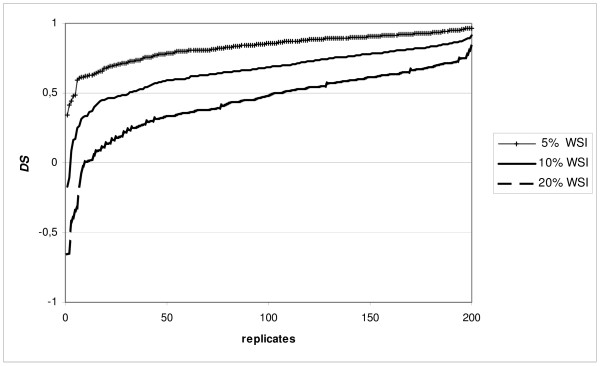
***DS *for 200 replicates of a standard population having 5%, 10% and 20% of WSI**. *DS *is fraction of diversity saved by applying optimal contributions based on observed pedigrees having WSI (wrong sire information). 200 replicates were arranged in order of *DS *result for standard populations having 5%, 10% and 20% WSI.

A real population represents a single replicate, not the average over replicates. Therefore, variance among replicates was illustrated. Figure 3 gives the *DS *for all 200 replicates of the standard population with 5%, 10% or 20% of WSI, arranged in order of their value. The 20 replicates with the poorest results have far lower values than average, and this phenomenon was observed in all simulated scenarios with WSI. Therefore, with an OCS over 10%, populations run the risk of losing much of their diversity

Our results indicate a moderately negative influence of wrong parent information on genetic variation saved by means of OCS in panmictic (random-mating) populations. Our findings suggest that in a panmictic population with approximately 10 to 20% WSI, which is common in practice (Table 1), OCS would, on average, save more genetic diversity than equal contributions. In some cases, however, selection of parents by OCS might decrease diversity more than the application of equal contributions. Nevertheless, equal contributions do not have that risk. Note that in real populations, dam information may also be wrong.

### Missing parent information (MPI)

**Figure 4:**
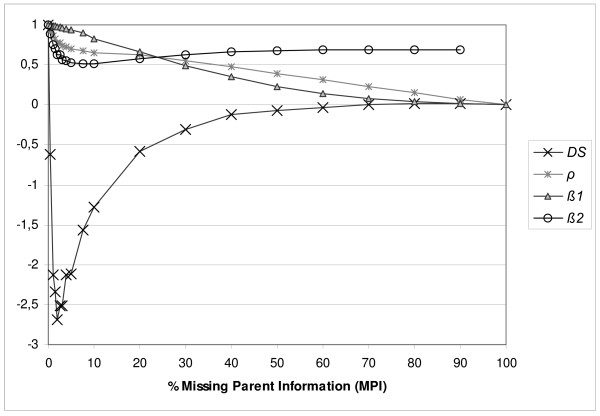
**Criteria in a panmictic population with missing parents**. Results are averages of 200 replicates of the standard population. Standard errors of results were 0.01 or less, except for *DS *where values up to 40% had standard errors up to 0.13.*DS *is fraction of diversity saved by applying optimal contributions based on observed pedigrees instead of true pedigrees. *ρ *is the correlation between observed kinship and true kinship.*β1 *is the regression coefficient of observed kinship on true kinship.*β2 *is the regression coefficient of true kinship on observed kinship.

Figure 4 gives *β1*, *β2*, *ρ *and *DS *of standard populations with different percentages of MPI. Though both parent records were set to missing, results for 'removal' of only one parent would show a similar pattern, since this single missing parent would miss both parents in the previous generation. True *N*_*EC *_and *N*_*OC *_exhibit the same values as in Figure 1 and are not shown. While *β1 *decreases almost linearly with an increasing percentage of missing parents, *β2 *immediately and strongly decreases towards 0.5 and then steadily returns to 0.7. This non-linear pattern of *DS *is even clearer. Even with very little MPI, *DS *exhibits a strong decrease and drops below zero, which is the value of diversity that would have been maintained if equal contributions were applied. From 3% onwards, *DS *gradually increases back to zero. At 100% *N*_*EC *_equals ${\tilde{N}}_{OC}$ and consequently *DS *is zero (equation 6). Finally, Figure 4 shows that correlation (*ρ*) is between *β1 *and *β2*, due to the relationship among *ρ*, *β1 *and *β2*. Note that although 1% missing parents already strongly affects diversity, the statistical criteria *ρ*, *β1 *and *β2 *do not elucidate this clear non-linear decrease of diversity. Thus, statistical criteria do not reveal the significance of the difference between true and observed kinships. A similar trend for *ρ*, *β1*, *β2 *and *DS *is observed in simulations with larger population sizes and differences in sex ratio (results not shown). In conclusion, simulations reveal a strong and non-linear effect on diversity due to missing parent information (MPI). The negative effect of MPI is best illustrated by *DS*. Even when as little as 0.5% of related animals without registered parents are treated as unrelated founders, OCS decreases diversity due to high contributions given to these animals or their offspring.

**Figure 5:**
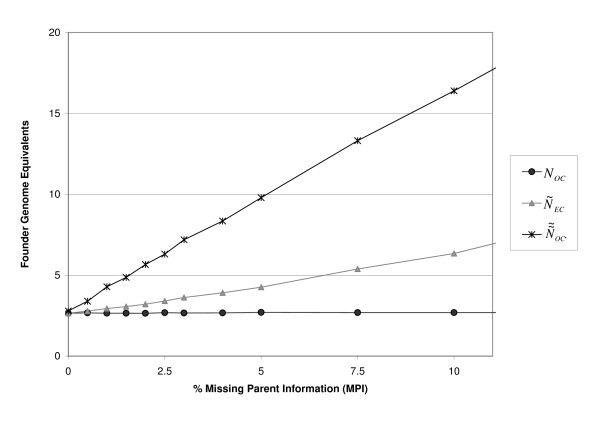
**Observed average and optimal kinship with different percentages of missing parents**.

To illustrate the overestimation of diversity due to MPI, Figure 5 shows the average FGE of true kinship (*N*_*ec*_), observed kinship (${\tilde{N}}_{ec}$) and observed optimal kinship (${\tilde{\tilde{N}}}_{OC}$) for the standard population with increasing MPI. When MPI is undetected, related animals with missing parents are regarded as unrelated founders. Founders are defined as animals without parents that are unrelated to other founder animals. Therefore, MPI leads to overestimation of diversity. Figure 5 shows that ${\tilde{N}}_{ec}$ and ${\tilde{\tilde{N}}}_{OC}$ increase with increasing MPI, while true diversity *N*_*ec *_is much lower.

Overestimation of diversity is also shown by *β2 *(Figure 4). To avoid overestimation of the conserved genetic diversity, it is important that observed kinship is an "unbiased" predictor of true kinship, which requires that *β2 *equals one. In the case of WSI, *β2 *gradually decreases. The strong decrease of *β2 *in the case of MPI indicates that the amount of conserved genetic diversity will be overestimated when selecting the least related individuals based on observed kinship. Although *β2 *indicates overestimation (Figure 4), it does not predict the strong overestimation of ${\tilde{\tilde{N}}}_{OC}$ in Figure 5.

A similar trend for *DS *was observed in simulations where only sires were missing, though *DS *behaved slightly differently. Logically, correlation for missing sire information decreased less rapidly than with both parents missing (results not shown).

### OCS breeding scheme for multiple generations

Fraction diversity saved (*DS*) after five generations of breeding by OCS based on observed pedigrees gradually decreased with increasing percentages of wrong sires (WSI). With WSI of 0%, *DS *is 1 by definition; with 10%, *DS *was 0.73; and with 25%, *DS *was 0.43. *DS *decreased roughly by 0.022 with each 1% increase of WSI. Extrapolation showed that *DS *would be zero at around 46% WSI.

**Figure 6:**
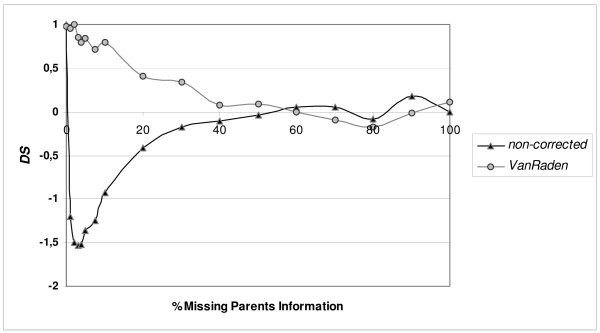
**Fraction diversity saved after five generations of breeding by OCS based on pedigrees having different percentages of missing parents**. *DS *(fraction diversity saved due to application of Optimal Contribution Selection, OCS) are averages of 200 replicates obtained after five generations of random breeding followed by five generations of OCS based on *non-*corrected or *VanRaden*-corrected kinship, calculated from pedigrees with different percentages of wrong sire information. Standard errors of results were 0.1 or lower.

Figure 6 shows *DS *for populations that were bred for five generations as the standard population followed by five generations OCS based on kinship calculated from pedigrees with different percentages of MPI. Once kinship was non-corrected as in Figure 4, and once kinship was corrected for missing pedigree information by VanRaden [19]. For non-corrected OCS, *DS *decreases strongly at levels as low as 0.5% MPI, and then drops below zero. From 5% missing parents onwards, *DS *increases again towards zero. For VanRaden-corrected OCS, *DS *starts at 1 and gradually drops to zero until 50% MPI. From 50% MPI and upward, on average no apparent difference is observed between equal contributions and OCS based on non- or VanRaden corrected kinship. Figure 6 shows again that OCS based non-corrected kinship calculated from pedigrees with MSI can only decrease diversity. Comparing Figure 6 with Figure 2, which shows results for a single generation, the decrease is not as strong as expected if all five generations were affected by MPI as strongly as a single generation. The reason for this is that the error did not accumulate each generation after it is 'incorporated' by OCS. Therefore relative loss due to pedigree errors mainly occurred in the first generation that started OCS.

This research investigated a panmictic population, assuming control over a population. In practice, species or populations differ in population structure due to aspects like unequal sex ratio and/or limited number of progeny per female, etc. Conservationists have to consider these constraints. With unequal sex-ratio for example, equal contributions cannot be applied and instead optimal management of mate selection across multiple generations yields lowest rates of increase of kinship [24,25].

## Conclusion

The results imply that using only pedigree information in conservation warrants caution.

On average, the genetic diversity saved by optimal contributions is less with low percentages of WSI. If WSI is over 35%, on average, optimal contributions preserve less genetic diversity than equal contributions. The impact of WSI on genetic diversity for a single population, however, might deviate from this average (Figure 3). In addition, when pedigrees are known to contain more than approximately 15% wrong parent information (misidentified fathers plus mothers) in a panmictic population, conservationist should consider alternative breeding methods, because expected gain is relatively low compared to alternatives like optimal management of mate selection across multiple generations. Populations in need of conservation, however, often deviate from a panmictic population. Furthermore, the type of error expected should also be taken into consideration. This research investigated the worst type of WSI. In practice, misidentified sires are sometimes related to the true sire, for example with natural mating occurs within herds. We also found that *DS *decreased slower due to VanRaden-corrected MPI (Figure 6) than due to WSI (Figure 4). In conclusion, wrong parent information above 15% might be acceptable in practice, depending on the type of error and the population structure. Traditionally, MPI is bypassed in pedigree analysis by the assumption that animals with unknown parents are founders [1], resulting in an overestimation of the available genetic diversity. Optimal contributions are extremely sensitive to differences in kinship between candidates. Small differences in pedigree can make the difference between significant or zero contribution for an individual animal. Animals with gaps in their pedigree will be considered unrelated and therefore be given high contributions. In this situation, equal contributions to each candidate parent would maintain diversity. Therefore, optimal contributions based on pedigrees with MPI can perform less well than equal contributions.

Overall this indicates that low percentage of MPI should always be corrected prior to the application of OCS. Even a simple correction of MPI by randomly assigned parents would increase diversity, which would leave breeders with wrong parent information. However, to correct for gaps in pedigrees, more sophisticated solutions have been presented. Ballou and Lacy [1] have proposed the calculation of kinship based only on the portion of the genome that descends from true founder animals, excluding the proportion due to animals with unknown parents. VanRaden [19] corrected gaps in pedigrees by assuming that unknown parents are related to all other parents by twice the average inbreeding level of that period. VanRaden is occasionally applied to calculate kinship [26]. Compared to VanRaden, the Ballou and Lacy-correction creates more variance among kinship values, which has a possible negative impact on OCS. Therefore, the VanRaden was applied to correct for MPI in this research.

We recommend two policies for conservation. First, measures that avoid errors in pedigree are encouraged. One obvious measure is to sample animal tissue, since DNA can be used both for parentage analysis and kinship estimation [27]. Second, pedigree-registration, like herd-books, should include information on the status of animals without parent records: whether they are (1) founders (wild-caught or otherwise known to be unrelated) or (2) related and descending from founders. Within kinship calculation, the latter should always be corrected, for example by using the VanRaden or a similar algorithm.

## Competing interests

The authors declare that they have no competing interests.

## Authors' contributions

PA conceived the study and carried out the simulations. PB participated in its design and coordination and helped to draft the manuscript. All authors read and approved the final manuscript.
